# Intracavernous Injection Therapy as Second-Line Treatment for ED After Radical Prostatectomy: A Literature Review

**DOI:** 10.3390/medicina62010111

**Published:** 2026-01-04

**Authors:** Adrian George Vlaicu, Cristian Mirvald, Salam Najjar, Radion Garaz, Igor Tsaur, Ioanel Sinescu, Cristian Surcel

**Affiliations:** 1 Department of Urology, “C.F.2” Hospital, 011464 Bucharest, Romania; 2 Faculty of Medicine, Carol Davila University of Medicine and Pharmacy, 050474 Bucharest, Romania; cristian.mirvald@umfcd.ro (C.M.); ioanel.sinescu@umfcd.ro (I.S.); cristian.surcel@umfcd.ro (C.S.); 3 Center for Uro-Nephrology and Renal Transplantation, Fundeni Clinical Institute, 022328 Bucharest, Romania; 4 Nord Hospital, 010948 Bucharest, Romania; salam.najjar@drd.umfcd.ro; 5 Department of Urology, University Hospital Tübingen, 72076 Tübingen, Germany; radion.garaz@med.uni-tuebingen.de (R.G.); igor.tsaur@med.uni-tuebingen.de (I.T.)

**Keywords:** erectile dysfunction, intracavernous injections, radical prostatectomy

## Abstract

Erectile dysfunction (ED) is a frequent and significant complication of radical prostatectomy that impacts patients’ quality of life. Intracavernous injection treatment has become an important approach to post-prostatectomy ED, providing an immediate and proven means of regaining erectile function in men who are unresponsive to oral phosphodiesterase type 5 inhibitors. The scope of this article is to discuss the mechanisms, efficacy, and safety of ICI treatments using vasoactive compounds as a second-line treatment for ED after radical prostatectomy. We consider clinical results, satisfaction, and compliance, as well as predictors of success, like nerve-sparing procedures. Through our interpretation of evidence and clinical experience, ICI therapy is a cornerstone of penile rehabilitation and ED management following radical prostatectomy. Also, the prospects for further treatment optimization and patient outcomes are discussed.

## 1. Introduction

Erectile dysfunction (ED) is a common consequence after radical prostatectomy (RP), with prevalence rates varying widely from 30% to 85%. This range is influenced by factors such as surgical technique and patient features. Studies found that 85% of men had ED 4.3 years post-RP, consistent with other high-prevalence findings [1]. In contrast, rates as low as 30% have been observed, especially when the nerve-sparing technique is used. The incidence rates vary from 12% to 96%, often higher in multicenter studies. Such variability shows the importance of study design in understanding ED post-RP [2].

Erectile dysfunction deeply affects men’s quality of life, impacting self-esteem, increasing stress, and creating relationship difficulties. Men with ED often experience low life satisfaction, feeling inadequate, and anxious about their sexual performance and masculinity. This distress can extend to their partners, who may also feel dissatisfied with changes in sexual frequency and intimacy levels. Although some partners initially adjust, many express disappointment over the reduced sexual activity, straining the relationship. Addressing ED effectively often requires supporting both partners to alleviate these emotions, aiming to restore intimacy and improve satisfaction for both individuals [3].

Standard practice now suggests PDE5 inhibitors as a first-line treatment for ED but concedes that they are unreliable after RP [4]. Success rates are based on nerve-sparing techniques during surgery, age, and preexisting medical conditions. PDE5 inhibitors do improve erections in most post-RP patients, but many have poor long-term results; therefore, complementary treatment is needed. About 30–35% of patients are non-responders to PDE5 inhibitors [5]. Second-line therapies are usually intracavernous vasoactive agents (e.g., prostaglandin E1) for non-responders (nR) to PDE5 inhibitors. These are injections that deliver the agent directly to the penile tissue, increasing the flow of blood and providing a solid erection. Intracavernous injections provide a predictable, rapid response in contrast to PDE5 inhibitors, which take longer to show results. This therapy is especially helpful for patients who cannot achieve acceptable intercourse even at maximally tolerated PDE5 inhibitor dosages. When trained in how to use and how to identify side effects, patients typically experience better rigidity and more successful sexual intercourse with intracavernous injections [6].

## 2. Materials and Methods

This article is a narrative, non-systematic review, intended to provide a synopsis of the existing evidence on the role of intracavernous injection (ICI) therapy as a second-line therapy for erectile dysfunction (ED) after radical prostatectomy. A focused, structured literature search of PubMed/MEDLINE, Scopus, and Web of Science databases from 1989 to 2025 used combinations of the following search terms: “intracavernous injections,” “intracavernosal therapy,” “erectile dysfunction,” “radical prostatectomy,” “PDE5 inhibitors,” “nerve-sparing,” and “priapism.” Articles were then included based on their relevance to post-prostatectomy ED and ICI therapy. Exclusion was non-systematic, broad, and included case series of <5 patients, lack of full-text availability, non-English language (when not translatable), and studies in PDE5 inhibitor responders only. Reference lists of included publications were searched for additional relevant articles.

After duplicates were removed, two authors performed an independent, unblinded search for relevance. There was no formal disagreement process, but discrepancies were settled by discussion. As this is not a systematic review, no PRISMA protocol or bias assessment was performed, nor was there a quantitative synthesis.

## 3. Results

### 3.1. Introduction of Intracavernous Injection Therapy

Intracavernous injections (ICI) of prostaglandin E1 (PGE1) have, for many years, been an effective treatment option for men with ED, and for whom the PDE5 inhibitor does not provide adequate response or is not tolerated well due to side effects. ICI safety and efficacy have been documented via patient reports, and a significant percentage (up to 62%) had satisfactory erections suitable for penetration. In studies, reports cited that approximately 56.9% of patients reported consistent erections sufficient for penetration, and an additional 23.2% achieved this more than half the time. On the safety matter, while reported pain levels, both at injection as well as erection, are mild (on an average of 2 out of 10 on an established pain scale), the low incidence of severe adverse effects, and the ease with which it can be self-administered further contribute to its safety record. Patients’ satisfaction with ICI is high, with the majority (>80%) of patients stating that the treatment met their expectations, both with respect to efficacy of erections and ease of use, and improved sexual life (81.7% of patients found that their erections were no more difficult to achieve with ICI than with other methods) [7]. More than 75% of patients also commented that their partners were satisfied with the ICI treatment [8].

### 3.2. Intracavernous Injection Therapy for ED: Mechanisms and Medications Used

Intracavernous injections consist of vasodilators being directly injected into the cavernous body of the penis, resulting in smooth muscle relaxation and engorgement of the corpora cavernosa with blood. The vasodilators are deposited locally; therefore, they do not pass into systemic circulation. Prostaglandin E1 (alprostadil) causes vasodilation by binding to specific prostaglandin receptors, which, through G proteins, activate the enzyme adenylate cyclase. The resulting increase in cAMP activates PKA, which decreases intracellular calcium concentrations, leading to relaxation of the smooth muscle. Evidence supports that careful dosing combined with patient education makes this treatment effective for erectile dysfunction. Clinical evidence for the use of alprostadil in ED shows a positive effect in 59–78% of men in terms of improved sexual activity with intraurethral preparations, and clinically meaningful benefit from topical forms. Alprostadil is one of the most widely used monotherapies in men with ED, among those who have not responded to a PDE5 inhibitor [9,10].

Papaverine and phentolamine act synergistically by improving smooth muscle relaxation in the context of penile cavernosal tissue. Papaverine is a smooth muscle relaxant that facilitates increased cyclic AMP and cyclic GMP in the intracellular compartment of smooth muscle, leading to vasodilation. Administration of phentolamine reduces the adrenergic input that exerts vasoconstrictive effects on smooth muscle, thereby reducing resistance to relaxation. The combination of these two drugs helps to improve smooth muscle relaxation beyond the action of the drugs administered individually because, by blocking the alpha-adrenergic receptor that promotes constriction of smooth muscle, phentolamine alters the microenvironment for relaxant activity, thus improving the action of papaverine on cyclic nucleotides. With improved inhibitory activity on smooth muscle, vasodilation will be improved, and erectile function is likely to be enhanced [11].

Combining agents in the treatment of severe ED maximizes efficacy due to the presence of various underlying mechanisms that contribute to the same condition. This is why, in combination with the abovementioned approaches, one agent may enhance vascular flow, another may deal with hormonal imbalances, and a third with psychogenic factors, potentially leading to better outcomes than monotherapy. The risk of side effects with combination approaches and the need to modulate the doses of every drug in combination therapy need to be considered [12].

### 3.3. Challenges of Achieving Spontaneous Erections Post-RP

Erectile function following RP is based on rehabilitating neurovascular systems, particularly the cavernous nerves and blood vessels necessary to produce and sustain erections. In non-nerve-sparing RP, structural and functional neurovascular injury disrupts important nerve communication and limits the potential of PDE5 inhibitors that rely on healthy nerves to produce nitric oxide (NO). Nerve-sparing RP is therefore key to optimizing erectile function recovery and improving the efficacy of pharmacotherapy. The nerve-sparing method is meant to minimize damage to bundles of neurovascular material essential for erections, but success is variable. With trained surgeons, bilateral nerve-sparing RP results in 5-year erectile function restoration of up to 40% versus 15% with unilateral or non-nerve-sparing RP [13]. Methods such as the “Veil of Aphrodite”, based on the patient’s anatomy, provide precise dissection and nerve preservation. Robotic surgery has the potential to raise 12-month potency to 70–80%, although success rates vary with patient age, health, and preoperative erectile function [14]. Treatment options for erectile dysfunction are presented in Table 1.

### 3.4. Need for Intracavernous Injection Therapy

PDE5 inhibitors work by enhancing the effect of nitric oxide (NO), which is released by nerve endings. After nerve injury, NO is not available in significant amounts. Preclinical studies in animals have found that PDE5 inhibitors may decrease oxidative stress and help in smooth muscle sparing, but this has not been seen in humans. In patients who have nerve-sparing surgeries, there may be an early benefit, but complete return of erections is rare. Vasoactive agents injected directly into the penis can trigger erections by increasing arterial blood flow and preventing venous outflow because they bypass the requirement for residual neural control. Papaverine is an agent that directly causes smooth muscle relaxation, and phentolamine is an alpha-adrenergic blocker, which causes dilation of arteries that lead to increased arterial inflow. Intracavernous injections serve as an alternative treatment approach for erectile dysfunction by functioning independently of normal neuronal pathways [15,19].

### 3.5. Efficacy of Intracavernous Injection Therapy in PDE5 Non-Responders

Intracavernosal injection (ICI) therapy has been demonstrated to be effective in treating erectile dysfunction (ED) in post-RP men, especially if PDE-5 inhibitors are ineffective. In one study of 117 men (mean age 65, and 4.1 years post-surgery), sexually active men using ICI enjoyed significant gains in erectile function, with significant improvements in the IIEF compared with their pre-ICI scores. In sexually active men using ICI after radical prostatectomy, IIEF-EF scores improved from 16.0 ± 6.9 to 20.8 ± 4.1 (*p* = 0.008). ICI therapy provided sufficient rigidity for penetration despite the loss of nitric oxide (NO), which was often compromised after nerve damage. Reasons for treatment discontinuation included dissatisfaction, injection-induced pain, and spontaneous resolution of erections [20]. Patient satisfaction can vary, and challenges with perceived ineffectiveness and discomfort affect adherence. ICI therapy remains a valuable second-line therapy for ED in post-RP patients, yet personal factors will often dictate the long-term acceptability of this treatment modality.

The use of intracavernosal injection therapy over a long period of time might lead to changes in patient responses, such that the injections do not work as well as they used to, leaving patients dissatisfied. Other reasons for discontinuation, including inadequate rigidity, discomfort from the injections, and loss of spontaneity, might also contribute to a perceived decline in effectiveness over time. In addition to changes in effectiveness and discontinuation, other factors might impact satisfaction with the treatment. In one long-term study, researchers found that those patients who remained on the therapy throughout the study reported greater satisfaction than patients who discontinued. This suggests that ongoing use might help patients maintain effectiveness and satisfaction with the treatment, while withdrawal from the therapy could lead to a perceived reduction in efficacy. Overall, while ICI can be enormously helpful initially, patients’ responses might change over time, and monitoring and adjusting treatment doses might be necessary to keep the treatment as effective for patients as possible over the long term [21].

### 3.6. Safety, Tolerability, and Patient Satisfaction with Intracavernous Injections

Examples of typical side effects include pain at the injection site, bruising, and fibrosis. Pain is a commonly reported side effect and is considered a major factor in treatment discontinuation. Patients may have experienced some type of pain with the injection, which then caused anxiety with the next one and made them feel reluctant to perform the procedure. Bruising is another side effect, which could occur because needle penetration into the skin creates injury to blood vessels, leading to bleeding and leakage to the skin, leaving a black and blue appearance, which may not be aesthetically pleasing and can make the person feel reluctant about having this form of treatment. Fibrosis or formation of scar tissue over time may also occur with ICI, especially if the site is not rotated properly or if the same site is used repeatedly, leading to a decrease or lack of efficacy of injections and further pain or discomfort. All the above can lead to compliance issues for the treatment [16].

Prolonged erections and the associated ischemia in priapism can lead to progressive corporal fibrosis and permanent erectile dysfunction if untreated. Before ICI therapy, clinicians must counsel the patient or partner thoroughly on the indications to recognize and report priapism (an erection lasting > 4 h associated with pain or abnormal perineal warmth, or an erection lasting > 6 h without pain or perineal warmth). Prior to allowing a patient to self-administer an ICI at home, dose titration of ICI medications must be done with serial monitoring of the effects in the office. If priapism does occur, corporal irrigation and injection of phenylephrine are immediate options, and surgical options may be necessary for more prolonged priapism or recurrent episodes. Early treatment is essential to prevent complications and preserve erectile function [22].

There are many reasons patients drop out of injections, including pain of the injections, anxiety over self-injection or needles, worry about side effects, perception that treatment is inefficacious, cost of the medication, scheduling hassles and inconvenience of follow-up, high level of non-spontaneity with sexual activity due to the treatment regimen, spouse or other partner resistance or lack of support, perception of artifice related to the treatment, difficulty acquiring the supplies necessary for auto-injection, and psychological issues associated with the trial dosing period such as stress or frustration [23]. Droput rates in studies involving intracavernous injections are presented in Table 2.

Across studies, dropout rates varied widely (4.9–64.3%), largely reflecting differences in follow-up intensity, ICI formulation used, patient training, and population characteristics. The most consistently reported reasons for discontinuation were injection-related pain, anxiety about self-injection, perceived lack of spontaneity, inadequate rigidity, and logistical inconvenience. Studies with structured follow-up and single-agent protocols (e.g., Alexandre et al.) reported markedly lower dropout rates, highlighting the importance of patient education and support in maintaining long-term adherence.

### 3.7. Critical Appraisal of Intracavernous Injection Evidence in Post-Radical Prostatectomy Patients

As with preoperative patients, data for the use of ICI in post-RP patients are limited and of variable quality. Domes et al. (2012) [20] found significant improvement in IIEF-EF score and penetration rigidity in a series of 117 post-RP men. However, the single-center design, open-label format, and high (nearly 50%) dropout rates limited the ability to interpret the long-term efficacy of ICI post-RP. Alexandre et al. (2007) [8] reported on a larger number of post-RP men in a multi-institutional retrospective study; however, men with general ED were not excluded, and therefore the observed outcomes, based primarily on patient satisfaction rather than objective measures, could not be extrapolated with certainty to the post-RP population only. The limited number of patients and diverse baseline characteristics, alongside short follow-up durations in multiple studies, constrain the ability to compare results. Patient adherence to therapy remains an issue with dropout rates of 46–64% secondary to penile discomfort, inconvenience of injections, and loss of spontaneity. Predictors of response to ICI have not been consistently reported in the post-RP population but may include bilateral nerve-sparing and early initiation of therapy [8,20].

### 3.8. Comparison with Other Second-Line and Third-Line ED Treatments

Looking at vacuum erection devices (VEDs) and self-injection therapy for erectile dysfunction, there are several factors to consider: invasiveness, ease of use, and efficacy. VEDs can be complicated to learn to use, depending on the level of impairment in manual dexterity of the individual, but partners can be easily trained. Self-injection therapy is considered invasive, and some men experience anxiety or pain over the procedure. Some men find the penis device awkward to use, especially in newer sexual relationships. Self-injection is often a more straightforward procedure, especially for men familiar with taking injections (e.g., insulin-dependent diabetics). Efficacy of both treatments is well-documented in the literature; dropout rates for VEDs are generally lower than self-injection, although this may be related to higher discomfort and invasiveness of self-injection [17,24].

Penile prosthesis is considered a third-line treatment for severe erectile dysfunction (ED) or ED that has not responded to first or second-line therapies such as oral medications or intracavernosal injections. Although penile implantation can be done safely, as with any type of surgery, these procedures may have complications that include infection, mechanical failure of the device, skin erosion or extrusion, and anesthetic issues. However, the long-term patient and partner satisfaction reported for penile implants remains quite high, with several studies reporting satisfaction rates of 92% for patients and 91% to 95% for their partners. Some of the high satisfaction rates reported for penile implants are due to the reliability of the device and its ability to function without special preparation [18].

Oral PDE5 inhibitors (sildenafil, vardenafil, tadalafil, and avanafil) tend to be preferred for ease of use and effectiveness and are usually more popular than alternatives such as vacuum erection devices or intracavernous injections. However, a considerable proportion of men with ED discontinue their ED therapies shortly after initiation, meaning that satisfaction alone is not determined by efficacy. Other considerations of patient preference are determined by the pharmacokinetic properties of the medications. For example, patients typically want the onset to be rapid, preferably in 15 min, and the duration to be 6 to 12 h, to allow spontaneity for sex. Moreover, when the partner is not satisfied, there can be implications for men’s perceptions of their treatment. Psychosocial factors such as self-esteem and anxiety play a role in overall satisfaction. All these factors require a nuanced understanding to optimize the management of ED and to ensure patient adherence [25].

### 3.9. Are New Effective Options on the Horizon? Emerging and Investigational Therapies

Stem cell therapy is a promising approach to the regenerative treatment of nerve and vascular dysfunction after RP and post-RP-ED. Surgical trauma is a causative factor for apoptotic cell death with cellular injury affecting a wide range of cell types. BM-MNCs (Bone Marrow-Derived Mononuclear Cells) are a heterogeneous population of cells that includes mesenchymal stem cells (MSCs) and endothelial progenitor cells (EPCs), which play a role by supplying agents to decrease cell death and by releasing growth factors, contributing to protection against cellular death, and preserving the integrity of the smooth muscle and endothelial cells that play a role in obtaining optimal penile function. Moreover, administration of BM-MNCs leads to increased levels of neuronal nitric oxide synthase (nNOS) and endothelial nitric oxide synthase (eNOS), two important enzymes for neurogenic and vasculogenic processes, respectively. In addition to addressing structural damage, this therapeutic option may also boost the neurotrophic and angiogenic reaction required for erectile function recovery. While standard pharmacotherapies for post-RP-ED fail in the most severe cases, stem cell therapy targets underlying cellular damage, potentially improving, and in some cases, increasing the quality of life for patients and reducing the need for more invasive treatments. However, research is needed to confirm the safety and effectiveness of these novel therapies [26,27].

Early data indicate that platelet-rich plasma (PRP) works by reversing erectile dysfunction through tissue repair. Preclinical studies showed that PRP can ‘repair’ erectile tissue via vasculogenic, neuroprotective, neurotrophic, reparative, and anti-inflammatory effects, albeit they did not evaluate erectile function directly. Efficacy data from clinical trials, even if of small numbers, including randomized controlled trials, have reported significant increases in IIEF score over baseline. In addition, the patient’s questionnaire scores suggest that there is a significant improvement in the quality of erections. Although minor side effects such as pain and transient bruising have been reported, more worrisome effects, such as small fibrotic plaques, have only occurred in a small number of cases. To date, the evidence base remains sparse, hindering our understanding of the mechanisms of action of PRP and the development of a consensus on standardized protocols for its use to treat ED [28].

It is suggested that, by enhancing blood flow as well as the outgrowth of regenerating nerve fibers, low-intensity extracorporeal shockwave therapy (LI-ESWT) improves erectile function after radical prostatectomy (RP) via at least the following three mechanisms. First, by inducing angiogenesis, it promotes the regeneration of tissue and the growth of new blood vessels. Second, by increasing the expression of growth factors and endothelial NO synthase through the recognition of cellular pathways, shockwaves may induce angiogenesis, thereby improving the blood flow to the penile tissue. Third, it may have reparative effects on the endothelial cells and nitric oxide synthase-producing nerves of the corpus cavernosum. The reasons for post-RP erectile dysfunction are multifactorial; shockwave therapy partly helps restore the physiological functions of the erectile tissue and the nerve fibers that come from the cavernous nerves, both of which are often compromised after RP [29].

### 3.10. Limitations and Gaps in the Current Literature

The literature does not provide much long-term data on the safety and efficacy of intracavernous injections for post-RP patients. Much of the current research has failed to provide critical longitudinal data on the long-term safety and efficacy of ICI in the prophylaxis of erectile dysfunction. One more obvious issue that limits the interpretation and generalizability of limited study populations is sample size, which can introduce a biased baseline. A study with a limited number of patients cannot represent the population from which those patients come, and the results can appear to be non-representative and biased compared with the larger population. Larger, more diverse studies must be carried out for the validity and applicability of any findings, particularly when the issue concerns a treatment that the patient might consider and undergo [30,31].

## 4. Conclusions

ICI therapy still has a place as an effective second-line therapy in selected men after RP, often due to its ability to circumvent nitric oxide-dependent cavernosal smooth muscle relaxation in the setting of neurovascular injury after RP and resulting in predictable rigidity. Compliance, counseling, and treatment of injection site pain are important in ensuring long-term satisfaction with this modality. Future directions in the medical treatment of ED after RP will include a more formalized rehabilitation strategy. Initiating ICI as part of a formal rehabilitation program, with closer follow-up, a standardized titration schedule, and partner training, may lead to better long-term adherence. However, these data are limited by small sample sizes, varying patient populations and methodology, and a lack of consistent and validated endpoints. Well-designed, prospective, placebo-controlled trials in patients with ED after RP only, with validated endpoints, like the IIEF-EF domain, will be needed to understand true efficacy and durability and allow for the identification of predictors of response. There are promising emerging therapies in the regenerative space that include stem cell-based therapies, PRP, and low-intensity shockwave therapy. These therapies have theoretical rationale and demonstrated biological efficacy but will need larger studies to determine safety, proper dosing, and clinical benefit. A multimodal approach to rehabilitation, combining the available effective medical, mechanical, and regenerative therapies, will likely be necessary to optimize patient outcomes.

## Figures and Tables

**Table 1:** Treatment options for erectile dysfunction post-radical prostatectomy.

Treatment Option	Mechanism of Action	Efficacy	Safety and Tolerability	Patient Satisfaction
PDE5 Inhibitors [15]	Enhance the nitric oxide pathway by inhibiting the enzyme PDE5, which leads to vasodilation and increased penile blood flow.	Often limited effectiveness after radical prostatectomy due to nerve and vascular damage. Success rates vary depending on nerve sparing during surgery, patient age, and overall health.	Generally well-tolerated.	Can improve erectile function in many patients, but a significant proportion experience inadequate responses.
Intracavernous Injections (ICI) of Vasoactive Agents [16]	Directly introduce vasodilators into penile tissue, enhancing blood flow and producing a reliable erection.	Effective in treating erectile dysfunction, especially in PDE5 non-responders. Provides sufficient rigidity for penetration despite the loss of nitric oxide.	Generally safe, with mild pain levels reported. Side effects can include pain at the injection site, bruising, fibrosis, and risk of priapism.	High satisfaction reported, with the majority of patients stating that the treatment met their expectations.
Vacuum Erection Devices (VEDs) [17]	Create an erection by drawing blood flow into the penis using a vacuum.	Can improve blood flow temporarily, but long-term benefits are not well-established.	Non-invasive, but it can be awkward to use.	Dropout rates are generally lower than for self-injection therapy.
Penile Implants [18]	Surgically implanted devices that provide a reliable and permanent solution for erectile dysfunction.	High satisfaction rates for both patients and partners. Reliable and spontaneous erections.	Potential complications include infection, mechanical failure, skin erosion, and aesthetic issues.	Consistently reported as a very high-satisfaction option for severe erectile dysfunction.

Note: This table summarizes the information presented in the present article regarding various treatment options for erectile dysfunction following radical prostatectomy.

**Table 2:** Dropout rates in studies involving intracavernous injections (ICI).

Study	Sample Size (N)	ICI Protocol/Agent Used	Dropout Rate	Main Reasons for Dropout (as Reported)
Alexandre et al., 2007 [8]	596	PGE1 monotherapy; minimum 3-month treatment	4.9%	Mild pain, alternative therapy, high adherence attributed to structured follow-up
Alves et al., 2018 [16]	28	Alprostadil (dose titrated individually)	64.3%	Injection-site pain, difficulty with self-injection technique, poor rigidity
Althof et al., 1989 [23]	131	Mixed ICI regimens (PGE1 and combinations)	46%	Needle anxiety, pain, lack of spontaneity, partner-related reluctance
Domes et al., 2012 [20]	117	Bi-Mix or Tri-Mix	49%	Perceived insufficient efficacy, discomfort, preference for less invasive options
Turner et al., 1992 [17]	106	Standardized ICI initiation protocol	60%	Pain, inconvenience, psychological reluctance, issues with obtaining injections

## Data Availability

Not applicable.

## Abbreviations

The following abbreviations are used in this manuscript:

ED	Erectile dysfunction
RP	Radical prostatectomy
PRP	Platelet-rich plasma
VEDs	vacuum erection devices
ICI	intracavernous injections
